# An Hour-Specific Hybrid DNN–SVR Framework for National-Scale Short-Term Load Forecasting

**DOI:** 10.3390/s26030797

**Published:** 2026-01-25

**Authors:** Ervin Čeperić, Kristijan Lenac

**Affiliations:** 1 HEP Telekomunikacije d.o.o., Kumičićeva 13, 51000 Rijeka, Croatia; ervin.ceperic@hep.hr; 2 Faculty of Engineering, University of Rijeka, Vukovarska 58, 51000 Rijeka, Croatia; 3 Center for Artificial Intelligence and Cybersecurity, University of Rijeka, Radmile Matejčić 2, 51000 Rijeka, Croatia

**Keywords:** short-term load forecasting, hybrid model, neural networks, support vector regression, feature selection, weather data, power consumption

## Abstract

**Highlights:**

**What are the main findings?**

- The hour-specific hybrid DNN–SVR framework achieves a 13.5% lower forecasting error than the PatchTFT baseline.
- Integrating global numerical weather predictions reduces error by 29.2% compared to using local measurements alone.

**What are the implication of the main findings?**

- Decomposing daily prediction into hour-specific submodels proves more effective than unified modeling for capturing diverse intraday load regimes.
- Validated on a 17-year dataset, this hybrid architecture offers a robust, high-accuracy alternative to pure deep learning baselines for operational grid management.

**Abstract:**

Short-term load forecasting (STLF) underpins the efficient and secure operation of power systems. This study develops and evaluates a hybrid architecture that couples deep neural networks (DNNs) with support vector regression (SVR) for national-scale day-ahead STLF using Croatian load data from 2006 to 2022. The approach employs an hour-specific framework of 24 hybrid models: each DNN learns a compact nonlinear representation for a given hour, while an SVR trained on the penultimate layer activations performs the final regression. Gradient-boosting-based feature selection yields compact, informative inputs shared across all model variants. To overcome limitations of historical local measurements, the framework integrates global numerical weather prediction data from the TIGGE archive with load and local meteorological observations in an operationally realistic setup. In the held-out test year 2022, the proposed hybrid consistently reduced forecasting error relative to standalone DNN-, LSTM- and Transformer-based baselines, while preserving a reproducible pipeline. Beyond using SVR as an alternative output layer, the contributions are as follows: addressing a 17-year STLF task, proposing an hour-specific hybrid DNN–SVR framework, providing a systematic comparison with deep learning baselines under a unified protocol, and integrating global weather forecasts into a practical day-ahead STLF solution for a real power system.

## 1. Introduction

Accurate short-term load forecasting (STLF) is critical for the stable and efficient operation of electrical power systems, particularly as they evolve to integrate intermittent renewable energy sources and adapt to changing consumer demands. Predicting the electrical load with precision allows grid operators to manage energy generation, scheduling, and load dispatch, thereby reducing the risk of imbalances that could lead to economic losses or supply disruptions.

As the demand for electricity evolves with advancements in technology and changes in consumer behaviour, the complexity of forecasting also increases. Historically, STLF relied on statistical methods such as autoregressive integrated moving average (ARIMA) models [1,2,3], praised for their simplicity and effectiveness in capturing linear trends and seasonal patterns. Despite their success in specific scenarios, these methods face significant challenges when applied to large datasets, often resulting in reduced accuracy due to their limited ability to handle complex, non-linear relationships inherent in big data [4,5].

In recent years, a range of deep learning techniques have demonstrated remarkable success in STLF [6,7]. Long Short-Term Memory (LSTM) networks have proven effective for their ability to model temporal dependencies [8,9,10,11]. Hybrid models, such as combining Ensemble Empirical Mode Decomposition (EEMD) with LSTM, have been proposed to reduce data noise before prediction [12]. Other architectures, including Deep Belief Networks (DBN) [13] and recurrent or attention-based models with Dynamic Time Warping (DTW) [14], have also shown strong performance. While powerful, recurrent models like LSTMs can be computationally intensive due to their sequential nature [15,16,17,18]. To address this, Transformer-based architectures [19,20,21] have been adapted for STLF, including models such as the Temporal Fusion Transformer (TFT) [10] and MDS-Transformer [22], which leverage attention mechanisms to efficiently handle long-term dependencies [23]. Recently, PatchTFT, a Transformer-based model that processes time series as sequences of overlapping patches rather than individual time points, has achieved state-of-the-art results in long-term forecasting by capturing local temporal patterns more effectively [24].

A comprehensive review by Eren et al. [25] highlights the breadth of recent deep learning approaches and current research directions in STLF, while the survey by Hasan et al. [26] provides a state-of-the-art comparative overview of load forecasting methods across classical, machine learning, and hybrid paradigms, further underscoring the need for rigorously evaluated, practically deployable hybrid models.

Beyond the power systems domain, several studies have demonstrated that coupling deep neural networks with margin-based learners can be beneficial in nonlinear regression tasks. Achite et al. [27] showed that hybrid DNN–SVM architectures outperformed standalone DNNs and other machine learning models in predicting daily pan evaporation, suggesting that SVM-based output layers can improve generalization when applied to deep-learned features. From a more theoretical standpoint, Li and Zhang [28] proposed Deep Neural Mapping SVMs, in which a multilayer perceptron functions as an explicit kernel mapping for an SVM classifier formalizing the integration of an SVM at the network’s output stage. Collectively, these studies support the rationale for architectures wherein a deep network serves as a feature extractor and an SVM or SVR performs the final prediction.

Motivated by these insights and by the specific characteristics of national-scale STLF, this paper investigates a hybrid architecture that enhances a Deep Neural Network (DNN) by integrating it with Support Vector Regression (SVR). SVR, an adaptation of Support Vector Machines (SVMs) [29], is well-regarded for its robustness in regression tasks, particularly in high-dimensional feature spaces [30]. In the considered setting, the hybridization addresses a fundamental challenge: while DNNs excel with very large datasets and recurrent architectures can exploit temporal dependencies across heterogeneous hours, an hour-specific modeling strategy which decomposes the 24 h forecasting problem into specialized subproblems operates on moderate-sized samples where both deep feature learning and margin-based regression can contribute effectively. The DNN serves as a nonlinear feature extractor that maps raw historical load, meteorological, and calendar variables into a compact latent representation for each hour, while the SVR, operating on this hour-specific representation, provides a margin-based regression mechanism that is robust under the moderate sample sizes and complex nonlinearities characteristic of individual hourly patterns.

The proposed model employs a multi-stage process. First, a gradient-boost-based feature selection algorithm identifies the most predictive variables, streamlining the input space. Next, for each forecasted hour, a DNN is trained on historical data to learn underlying patterns specific to that hour. The activations from the penultimate layer of each hour-specific DNN, representing high-level features, are then used as input for an SVR model that performs the final regression. This architecture is particularly suited to complex datasets where external factors such as weather exert hour-dependent influences on consumption.

Furthermore, this study addresses the common challenge of limited or inconsistent local weather data by integrating data from global numerical weather prediction (NWP) models. Ensemble forecasts from the TIGGE archive are combined with local observations to construct consistent meteorological predictors for the Croatian power system, embedded into an operationally realistic day-ahead forecasting pipeline.

From a sensing perspective, the proposed framework constitutes a feature-level fusion of heterogeneous sensor data streams, combining grid-level load measurements, local meteorological observations, and global numerical weather prediction outputs.

The proposed approach is evaluated using Croatia’s electrical load data from 2006 to 2022. Specifically, this work aims to achieve the following:

1. Evaluate the forecasting performance of an hour-specific hybrid DNN-SVR approach against its constituent models and several deep learning baselines under a unified experimental protocol;
2. Assess the impact of varying training data volume on model accuracy by considering distinct historical periods within a 17-year national dataset;
3. Quantify the performance benefits of incorporating global NWP-based weather information into the STLF process for a real-world power system.

Instead of treating DNN-SVR hybridization as a mere substitution of the output layer or relying on a single hybrid model for the entire forecasting task, this study embeds the hybrid architecture within an hour-specific decomposition framework, enhanced by systematic feature engineering and multi-scale weather data assimilation to tackle a long-horizon national forecasting problem. Specifically, the contribution lies in the joint and systematic treatment of the following elements, which have not been previously combined in the STLF literature:

1. A 17-year, country-scale STLF problem (Croatia, 2006–2022) with a clearly defined, held-out test year, providing a realistic and challenging benchmark for evaluating long-term model performance;
2. A framework of 24 h specific hybrid DNN-SVR models, each trained on tailored feature sets and hour-optimized latent representations, enabling the capture of distinct intraday load regimes rather than forcing a single model to fit heterogeneous hourly patterns;
3. A comprehensive empirical comparison with strong modern deep learning baselines including feedforward DNNs, LSTM Seq2Seq with attention, and multiple Transformer-based architectures, including the Temporal Fusion Transformer and PatchTFT, under identical data preprocessing and evaluation protocols, ensuring a fair and reproducible assessment;
4. The operational integration of TIGGE-based global numerical weather prediction forecasts with local meteorological observations into a practical day-ahead STLF workflow, addressing the common challenge of incomplete historical weather data in real-world power system applications.

Taken together, these elements position the proposed framework not merely as an incremental modification of existing hybrid methods, but as a comprehensive, practically validated approach to national-scale STLF that combines architectural hybridization with strategic modeling choices and rigorous empirical evaluation.

## 2. Materials and Methods

This section outlines the data sources and engineering steps underpinning the proposed forecasting framework. The study is built around a long-horizon, country-scale dataset and an operationally realistic setup, which together form a central element of the contribution. We employ 17 years of national load measurements and meteorological information to construct features for an hour-specific hybrid DNN-SVR framework and all baseline models under a unified protocol.

### 2.1. Croatian Electrical Power Consumption Data (2006–2022)

The primary target variable is Croatian electrical power consumption, spanning the period from 2006 to 2022. The data are obtained from the ENTSO-E Transparency Platform [31] and represent country-level hourly load. This extended time horizon captures long-term structural changes in demand, economic cycles, and exceptional events, providing a realistic and challenging benchmark for STLF. All models evaluated in this study, including DNN, DNN-SVR, LSTM, Transformer, and the proposed hybrid, are trained and tested on this common dataset.

### 2.2. Temporal and Calendar Data in Load Forecasting

Temporal data are critical for capturing the cyclical nature of electricity demand. Calendar information was incorporated by categorizing holidays into three types (major holidays, pre-holidays, and other non-working days) and encoding weekdays (1–7) and months (1–12).

**Figure 1:**
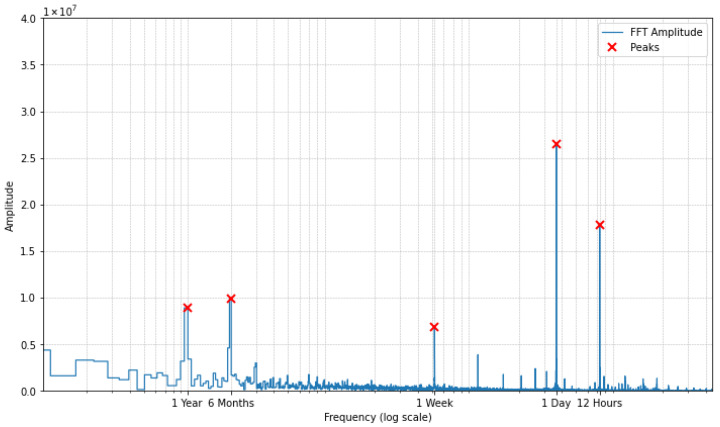
Frequency analysis of Croatian electrical load data (2006–2022) identifying dominant periodicities: yearly, semi-annual, weekly, daily, and semi-daily. These cyclic patterns inform the temporal feature engineering and hour-specific modeling strategy.

Frequency analysis of the historical load data (Figure 1) confirmed several key periodicities reflecting the cyclical nature of electricity consumption: yearly, semi-annual, weekly, daily, and semi-daily. To model this seasonality, for time-series models, trigonometric functions were used:

(1) $$\mathrm{Seasonal}\;\mathrm{component}=A\cdot \sin\left(\frac{2\pi t}{T}\right)+B\cdot \cos\left(\frac{2\pi t}{T}\right)$$

where *t* is the time index and *T* is the period of the seasonality (e.g., 24 for daily).

### 2.3. Integration of Global Weather Forecast Model

To address the challenge of sparse or inconsistent historical local weather forecasts, this study integrated data from a global weather forecast model. The TIGGE (The International Grand Global Ensemble) database [32], which amalgamates ensemble forecast data from 13 global NWP centers, was used as the source. Forecast data for the following parameters were utilized: 2 m temperature (2t), 2 m dewpoint temperature (2d), mean sea level pressure (msl), total cloud cover (tcc), total precipitation (tp), and 10 m U/V wind components (10u, 10v). Forecasts were generated for lead times of 0 to 72 h at 6 h intervals. Given the spatial resolution of global NWP models and the structure of the Croatian power system, the construction of weather-based predictors is focused on two major urban and climatic centres: Zagreb, representing the continental north, and Split, representing the coastal south. For each of these regions, multiple GRIB grid points in the immediate vicinity of the cities are selected to approximate local conditions and capture relevant mesoscale variability:

- Zagreb region: grid points surrounding approximately (${\mathrm{lat}}_{1}=45.5$, ${\mathrm{lon}}_{1}=16$) to (${\mathrm{lat}}_{2}=46$, ${\mathrm{lon}}_{2}=16$)
- Split region: grid points surrounding approximately (${\mathrm{lat}}_{1}=43.5$, ${\mathrm{lon}}_{1}=16.5$) to (${\mathrm{lat}}_{2}=44$, ${\mathrm{lon}}_{2}=15.5$)

For each variable, the values from these neighbouring grid points are used to form location-specific predictors that reflect the dominant continental and coastal weather regimes influencing national load. In addition, observed 2 m temperature measurements from meteorological stations in Zagreb and Split, obtained from Weather Underground [33], are included where available as supplementary features. These local observations provide an independent reference to the NWP-based predictors and enhance the robustness of the overall weather–load feature set.

### 2.4. Data Partitioning and Temporal Coverage

**Figure 2:**
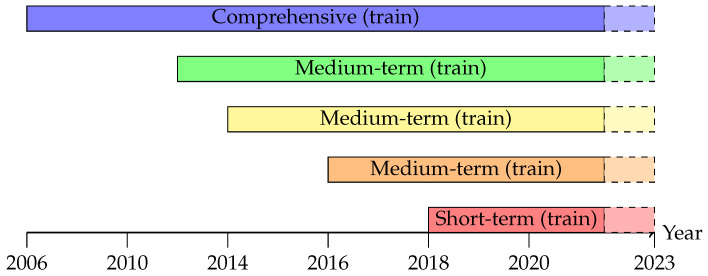
Temporal coverage of training datasets (solid bars, 2006–2021 development period) and held-out test set (dashed bars, 2022). Five training windows are evaluated: comprehensive (2006–2021), three medium-term periods (2012–2021, 2014–2021, 2016–2021), and short-term (2018–2021). All models use an 80/20 train–validation split within the development period and are tested exclusively on 2022.

The data from 2006 through 2021 are used for model development, while the entire year 2022 is reserved exclusively as a held-out test set for performance evaluation. This design reflects a realistic deployment scenario in which models trained on historical data must generalize to a future year with potentially different operating conditions (Figure 2).

Within each training period, 80% of the available samples are used for parameter estimation and 20% are reserved as a validation set. This validation split is applied consistently across all architectures (DNN, hybrid DNN-SVR, LSTM Seq2Seq + Attention, Transformer-based models), and is used for model selection and hyperparameter tuning without using any information from the 2022 test year.

To analyze the impact of training data volume on forecasting performance, several temporal subsets of the development period are considered:

- **Comprehensive period:** 2006–2021;
- **Medium-term periods:** 2012–2021, 2014–2021, 2016–2021;
- **Short-term period:** 2018–2021.

These subsets are used uniformly for all models to enable a fair comparison of how different architectures benefit from additional historical data.

### 2.5. Data Quality Control and Preprocessing

Given the 17-year operational dataset, particular care was taken to ensure consistency, avoid information leakage, and preserve physically meaningful variability.

#### 2.5.1. Missing Values and Consistency Checks

Hourly load data from ENTSO-E and TIGGE-based NWP forecasts were checked for completeness and temporal alignment and form an effectively continuous series over the study period. In contrast, occasional gaps in local observed temperature records (Zagreb and Split) are handled conservatively: any timestamp for which a required temperature value is missing is excluded from model training and validation, so incomplete input vectors are not used. No synthetic values are introduced into the held-out 2022 test set.

#### 2.5.2. Structural Changes and Temporal Integrity

Long-term demand shifts driven by economic conditions, efficiency measures, and the COVID-19 period are left explicitly in the data. Models are trained on chronologically ordered historical samples and evaluated on the unseen year 2022, without any back-looking from test into training. All resampling, feature construction, and TIGGE integration respect the time axis to prevent leakage.

#### 2.5.3. Scaling and Normalization

For all learning algorithms (SVR, DNN, hybrid DNN-SVR, LSTM Seq2Seq + Attention, Transformer-based models), normalization and scaling parameters are fitted exclusively on the training portion of each development set (the initial 80%) and then applied to the corresponding validation (20%) and 2022 test data. This protocol is used consistently across all models to ensure a fair and leakage-free comparison.

#### 2.5.4. Fusion of Heterogeneous Sensor Data Streams

From a sensing and data-engineering perspective, the proposed short-term load forecasting framework can be interpreted as a feature-level fusion of heterogeneous sensor data streams. Specifically, the model integrates (i) national-scale electrical load measurements obtained from the ENTSO-E Transparency Platform, (ii) local in situ meteorological observations from Weather Underground stations, and (iii) global numerical weather prediction (NWP) forecasts derived from the TIGGE archive. Each of these data sources represents a distinct sensing modality with different spatial coverage, temporal resolution, and uncertainty characteristics, providing complementary information for the forecasting task.

The fusion of these heterogeneous streams introduces several practical challenges that must be addressed during data preparation and input construction. First, temporal heterogeneity arises due to mismatched sampling frequencies, with load data available at hourly resolution, TIGGE forecasts provided at six-hour intervals for multiple lead times, and local station measurements exhibiting irregular reporting patterns over the 17-year study horizon. Second, spatial heterogeneity must be addressed, as point-based meteorological stations capture local microclimatic effects, whereas global NWP products represent area-averaged atmospheric conditions on a coarse spatial grid. Third, long-term sensor availability and continuity cannot be guaranteed: local meteorological stations may experience outages, reporting gaps, or operational changes over multi-year periods, resulting in incomplete or inconsistent observational records. Such issues are well documented in heterogeneous sensor networks across diverse application domains and necessitate robust fusion and quality-control strategies to avoid propagating artefacts into downstream predictive models [34].

To address these challenges, a conservative and operationally realistic fusion strategy is adopted. All data streams are first temporally aligned to a common hourly timeline. Forecast variables available at coarser temporal resolution are mapped to hourly predictors without introducing artificial high-frequency variability. Missing values in local meteorological observations are handled by excluding incomplete feature vectors from training and validation, while preserving a fixed input structure and ensuring that no synthetic or imputed values contaminate the learning process or the held-out test set. Redundancy between global NWP forecasts and local meteorological observations is deliberately exploited at the data source level over the full study horizon, reflecting the coexistence of observational and forecast-based representations of atmospheric conditions derived from independent sensing mechanisms. While samples with missing local observations are conservatively excluded from training and validation, the availability of physically consistent NWP-based predictors ensures that retained samples remain informative and robust across the full study horizon. The resulting feature-level fusion yields a unified, quality-controlled input representation that preserves the complementary strengths of global forecasts, local sensors, and grid-level load measurements, while maintaining temporal integrity and physical consistency.

Beyond its role in improving forecasting accuracy, this fusion strategy aligns the proposed framework with broader principles of intelligent sensing systems, where robustness, redundancy, and the integration of heterogeneous data streams are essential for reliable operation. As such, the presented approach contributes not only to short-term load forecasting, but also to the wider field of sensor fusion-driven predictive analytics.

### 2.6. Gradient-Boost-Based Feature Selection

Feature selection (FS) is a critical component of the proposed forecasting framework and is applied consistently across all model types (SVR, DNN, and DNN-SVR) to derive informative and compact subsets of predictors from the full candidate set.

The adopted FS method is based on Extreme Gradient Boosting (XGBoost). An XGBoost regressor is trained on the development period (2006–2021) using the full set of input variables, and features with importance scores below a predefined threshold (0.002 in this study) are discarded. The resulting ranked subset is reused across all models to ensure consistency and reproducibility.

This work builds on prior STLF research using SVR [30], where a stepwise FS method was employed alongside PSO-based hyperparameter tuning. Although gradient boosting was not explored in that context, the stepwise approach demonstrated strong performance. In the present study, the transition to gradient-boost-based FS is motivated by its ability to capture nonlinear relationships, its built-in importance metrics, and its scalability to larger feature spaces. These properties make it particularly well suited for integration with deep learning and hybrid architectures, while also supporting a more automated modeling workflow.

## 3. Modeling Framework and Forecasting Model

This section formalizes the proposed forecasting framework: an hour-specific hybrid pipeline that couples a feedforward DNN as a nonlinear feature extractor with a SVR head as the final regressor. The design targets day-ahead STLF at hourly resolution by decomposing the task into 24 specialized pipelines (one per hour), while retaining the unified data-engineering and feature-selection path from Section 2.

### 3.1. Input Assembly and Gradient-Boost Feature Selection

Before introducing the hybrid DNN-SVR, we formalize the front end that prepares the hour-specific design matrix consumed by all models in this section. The front end has two stages: construction of a candidate input vector at the forecast issue time and gradient-boost filtering that yields a compact, informative subset of predictors.

At issue time t= 08:00 we forecast the next-day hours 00:00–23:00, i.e., horizons H={16,17,…,39}, hours ahead of *t*. In the hour-specific strategy we train *one independent pipeline per hour* h∈H. Each pipeline uses only features constructed for its own target hour, undergoes its *own* feature selection (FS), and learns its *own* DNN encoder and SVR head under the common data protocol of Section 2.

#### 3.1.1. Inputs per Hour *h* (Known at *t*)

For each h∈H we assemble a time-aligned design vector by concatenating the following:

- **Near-term history (fixed window).** A horizon-invariant window of recent observations for load and observed temperatures:
  $$K_{\mathrm{nt}}=\left\{0,1,\ldots ,32\right\}.$$
  We include $\left\{y_{t-\ell }:\ell \in K_{\mathrm{nt}}\right\}$ and $\left\{T_{t-\ell }^{\mathrm{ZG}},\;T_{t-\ell }^{\mathrm{SP}}:\ell \in K_{\mathrm{nt}}\right\}$;
- **Hour-conditioned weekly/near-week anchors.** Anchors are *relative to the predicted hour*: $\mathrm{anchor}\left(X,k,h\right)=X_{t-(k-h)}$. For **load** we use
  $$k\in \left\{72,96,120,144,168,336\right\},\;{\left\{\mathrm{anchor}\left(y,k,h\right)\right\}}_{k}.$$
  For **temperature** (Zagreb and Split) we use
  $$k\in \left\{72,96,120,144,168\right\},\;{\left\{\mathrm{anchor}\left(T^{\mathrm{ZG}},k,h\right),\;\mathrm{anchor}\left(T^{\mathrm{SP}},k,h\right)\right\}}_{k}.$$
  For all h∈{16,…,39} these indices refer to times ≤ *t*, and hence are fully known at inference.
- **TIGGE NWP block (hour-invariant).** Global NWP predictors for Zagreb and Split: 2 cities × 2 nearby grid points × 7 variables (2t, 2d, msl, tcc, tp, 10u, 10v) × 13 leads (0–72 h in 6 h steps), yielding 2×2×7×13=364 features per timestamp;
- **Calendar descriptors.** Three integer-coded variables: *holiday_type* ∈ {major, pre-holiday, other non-working}, *weekday* =1–7, *month* =1–12.

Timestamps lacking a required temperature value (Zagreb or Split) are excluded so that no incomplete vectors enter training/validation (cf. Section 2).

#### 3.1.2. Candidate Dimensionality (Before FS)

With the fixed window, the lagged scalars per hour are

$$d_{\mathrm{lags}}=\underset{\mathrm{load}\;\mathrm{near}-\mathrm{term}}{\underset{︸}{33}}+\underset{\mathrm{load}\;\mathrm{anchors}}{\underset{︸}{6}}+\underset{\mathrm{two}\;\mathrm{temp}\;\mathrm{near}-\mathrm{term}}{\underset{︸}{2\times 33}}+\underset{\mathrm{two}\;\mathrm{temp}\;\mathrm{anchors}}{\underset{︸}{2\times 5}}=115.$$

Adding the TIGGE block and calendar integers gives a constant size

$$d_{\mathrm{FS}}=\underset{\mathrm{TIGGE}}{\underset{︸}{364}}+\underset{\mathrm{lags}}{\underset{︸}{115}}+\underset{\mathrm{calendar}}{\underset{︸}{3}}=482$$

for every *h*. Table 1 provides a concise summary of the input feature categories.

#### 3.1.3. Feature Selection (Per Hour)

For each *h* we train an XGBoost regressor on the development period (chronological 80%/20% train/validation split) and prune predictors whose importance falls below 0.002. Per-hour FS reduces dimensionality and multicollinearity while preserving the strongest hour-specific meteorological, load, and calendar signals.

The feature selection approach differs by model type to reflect their architectural requirements: DNN and DNN-SVR use the full set of XGBoost-selected features (typically 30–40 features), including historical load, calendar variables, weather observations, and forecast covariates. In contrast, Transformer-based models and LSTM Seq2Seq variants use feature selection only on the forecast weather block, while directly including the full set of past load, temperature observations, and periodic calendar encodings without selection. This design ensures compatibility with their structured stream input format.

### 3.2. Hybrid DNN-SVR Architecture

**Figure 3:**
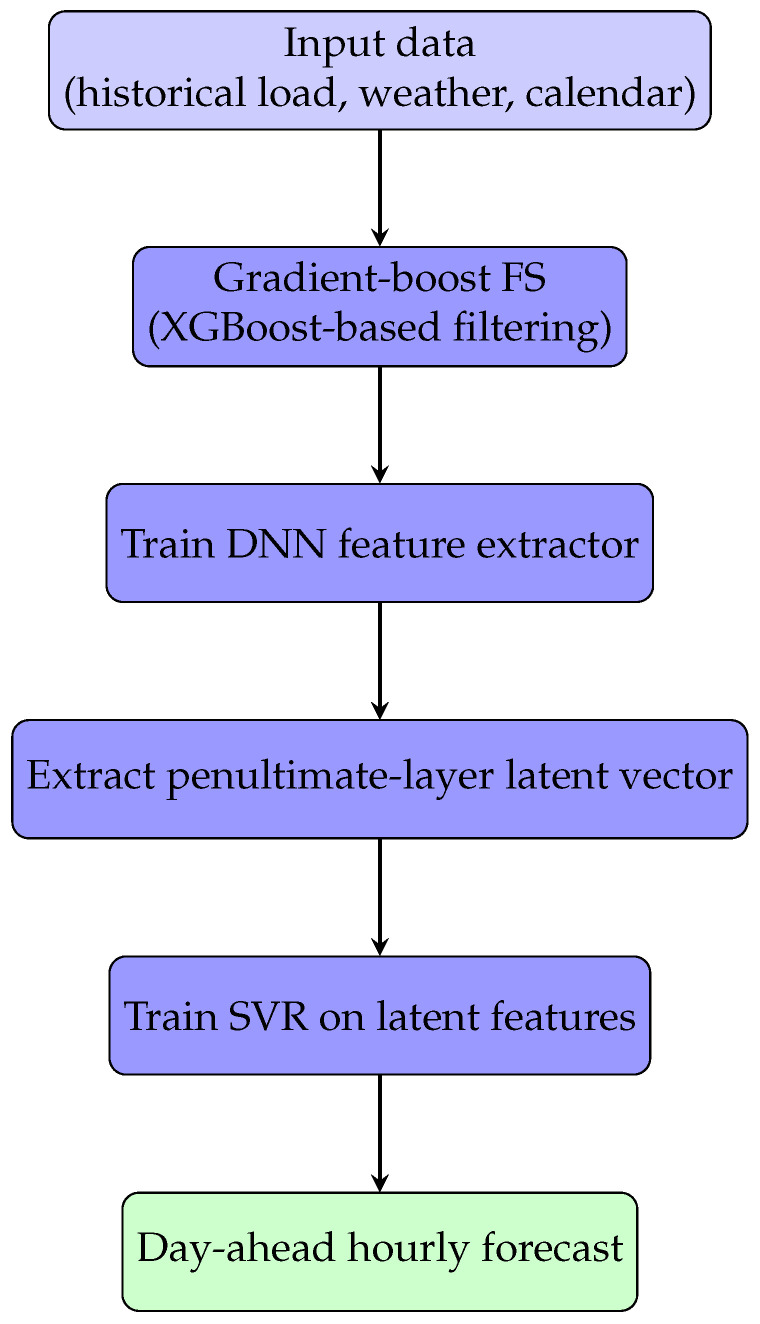
End-to-end workflow of the proposed hybrid DNN-SVR framework.

Building on Section 3.1, we introduce an hour-specific hybrid pipeline for day-ahead STLF in which a feedforward DNN encodes the filtered inputs into a compact latent representation and a SVR head (linear or RBF) performs the final margin-based regression. For each hour *h*, gradient-boost FS is applied *independently* to that hour’s candidate set (chronological 80/20 split), producing an hour-tailored subset that is then used by the DNN encoder and the SVR head for the same hour. The procedure is identical across hours, but the selected variables can differ, pairing the DNN’s representation power with the SVR’s sample-efficient, stable generalization. The workflow is summarized in Figure 3.

Operationally, the hybrid training proceeds in two stages. First, for each hour and training configuration, the DNN is trained end-to-end with a linear output layer using Mean Absolute Error (MAE) loss and the optimization setup described in Section 4. Once the DNN has converged, the final dense output layer is discarded and the activations of the penultimate layer are extracted. Second, an SVR model (linear or RBF kernel) is fitted on these latent features, with hyperparameters selected on the validation split. At inference time, a new input passes through the DNN to produce its latent representation, which is then fed to the trained SVR to obtain the final load forecast.

#### 3.2.1. Feedforward DNN Feature Extractor

The feedforward DNN forms the core nonlinear representation learner in the proposed framework and simultaneously serves as a standalone baseline model. All DNNs are implemented as multilayer perceptrons (MLPs) using TensorFlow (v2.9.1) and Keras (v2.9.0) [35,36].

The architecture comprises the following:

- An input normalization layer that standardizes the selected features;
- A sequence of fully connected (dense) hidden layers with Rectified Linear Unit (ReLU) activation, where the number of layers and neurons per layer is varied during architecture search;
- A final hidden layer whose activations constitute a compact latent representation of the input space.

For the standalone DNN baseline, this latent representation is followed by a single linear output neuron that directly predicts the target load. In the hybrid configuration, the same DNN is trained in an identical manner, but the final dense output layer is removed after training and the activations of the penultimate layer are used as input features for the SVR regression head (Section 3.2.2). In both roles, the DNN learns high-level nonlinear mappings from historical load, meteorological variables, and calendar features to an hour-specific latent space that is subsequently exploited either by a dense output layer or by SVR.

#### 3.2.2. Support Vector Regression Component

SVR, the regression counterpart of Support Vector Machines, is employed as the regression component in the hybrid architecture. All SVR models are implemented with the scikit-learn library (v1.2.0) and are trained on feature sets derived from the DNN-generated latent representations.

We consider both linear and Radial Basis Function (RBF) kernels. For the RBF kernel, the principal hyperparameters are as follows:

- The regularization parameter *C*, controlling the trade-off between model complexity and training error;
- The kernel coefficient γ, determining the effective influence range of individual samples in the feature space;
- The insensitivity parameter ε, specifying the width of the ε-tube around the regression function within which errors are not penalized.

Hyperparameters (C,γ,ε) are selected via systematic grid search evaluated on a 20% validation split from the development period, separately for each modeling strategy (single-model and 24-model setups), using only past data to avoid temporal leakage. Prior to training, all input features are standardized using StandardScaler from the scikit-learn library fit on the training subset and applied consistently to validation and test data, which is essential for stable SVR performance.

Within the hybrid framework, SVR operates on the low-dimensional representations produced by the penultimate DNN layer. The penultimate layer is specifically chosen because it captures the highest-level abstract representations learned by the DNN while maintaining a fixed dimensionality suitable for SVR input, a principle well established in transfer learning, where later layers encode task-specific features and earlier layers capture more generic patterns [37]. This design allows the kernel-based regressor to focus on margin-based regression in a compact, task-specific feature space, thereby quantifying and exploiting the added value of combining deep feature learning with SVR.

### 3.3. Unified Day-Ahead DNN-SVR Model (Hour-Conditioned, Single-Output)

In contrast to the 24-model strategy in Section 3.1, we also consider a *single* shared model that predicts one target hour at a time while reusing the same network across all horizons. Throughout this subsection the forecast is issued at t= 08:00 and the next-day targets are 00:00–23:00, i.e.,

H={16,17,…,39}

hours ahead of *t*.

#### 3.3.1. Input Design (Fixed Dimension, Hour-Conditioned)

For each requested horizon h∈H we build a time-aligned feature vector from four blocks, all known at *t*; definitions of variables and preprocessing follow Section 3.1:

- **Near-term history (constant window).** To provide a stable recent context without colliding with hour-conditioned anchors, we use the fixed window
  $$K_{\mathrm{nt}}=\left\{0,1,\ldots ,8\right\},$$
  applied to load *y* and observed 2 m temperatures for Zagreb and Split: $\left\{y_{t-\ell }:\ell \in K_{\mathrm{nt}}\right\}$ and $\left\{T_{t-\ell }^{\mathrm{ZG}},\;T_{t-\ell }^{\mathrm{SP}}:\ell \in K_{\mathrm{nt}}\right\}$;
- **Hour-conditioned weekly/near-week anchors.** Using the anchor definition from Section 3.1, $\mathrm{anchor}\left(X,k,h\right)=X_{t-(k-h)}$, we preserve weekly structure *relative to the target hour*. For load we take k∈{48,72,96,120,144,168,336}; for temperature k∈{48,72,96,120,144,168}. For all h∈{16,…,39} these indices are nonnegative, and hence observable at *t*;
- **TIGGE NWP predictors (hour-invariant).** We append the same TIGGE block as defined in Section 3.1, yielding 364 features per timestamp;
- **Calendar descriptors and horizon tag.** We use the same three integer-coded calendar variables as in Section 3.1 (no one-hot, no sin/cos) and add a scalar *target_hour* = h so the shared model is explicitly conditioned on the requested hour.

#### 3.3.2. Feature Selection and Scaling

Unlike the per-hour FS in the 24-model setup, FS is executed *once* on the pooled design (all h∈H) using the same XGBoost procedure and threshold as in Section 3.1; the retained subset is then reused for every horizon. All scalers are fit on training folds only and applied to validation and the held-out test year.

#### 3.3.3. Training and Inference

The unified model follows the same two-stage pipeline as above: a feedforward DNN encodes the FS-filtered inputs into a compact latent vector, and an SVR head (linear or RBF) performs the final regression. At inference, to predict hour *h*, we form the hour-conditioned input (including *target_hour* = h), pass it through the frozen DNN encoder, apply the training-fitted scaler, and obtain ${\hat{y}}_{t+h}$ from the SVR head.

## 4. Results

This section presents the performance analysis of the STLF models described in Section 3. All models were implemented in Python using the Keras library with a TensorFlow backend. The evaluation is designed to assess the effectiveness of the proposed hybrid model against baseline methods, compare different modeling strategies, and analyze the impact of feature selection, data size, and external weather data.

This section evaluates the proposed hour-specific hybrid DNN-SVR framework against its constituent models and deep learning baselines under the unified framework described earlier. The goals are to quantify the contribution of the hybridization relative to standalone DNN and SVR, study the effect of modeling strategy (single vs. 24-model), assess the impact of training data volume and evaluate the benefit of integrating forecast-based weather predictors.

All models predict the 24 hourly loads of the next day, with forecasts issued at 08:00, corresponding to a 16–39 h ahead horizon. The full year 2022 is used exclusively as an unseen test set. All models were implemented in Python 3.9.13 using TensorFlow 2.9.1 with the Keras 2.9.0 API, scikit-learn 1.2 for SVR and preprocessing, XGBoost 1.7.2 for feature selection, and NumPy 1.22.3.0/Pandas 1.4.4 for data manipulation. Experiments were conducted on a workstation equipped with an Intel (R) Core i5-10500 processor (six cores, 3.10 GHz base frequency) and 16 GB RAM.

### 4.1. Evaluation Metrics and Setup

Model performance was primarily evaluated using the Mean Absolute Percentage Error (MAPE), which quantifies the forecast error as a percentage of the actual value. It is defined as follows:

(2) $$\mathrm{MAPE}=\frac{100\%}{n}\sum_{i=1}^{n}\left|\frac{y_{i}-{\hat{y}}_{i}}{y_{i}}\right|$$

where *n* is the number of observations, $y_{i}$ is the actual value, and ${\hat{y}}_{i}$ is the predicted value. Additionally, MAE was used to measure the average magnitude of errors in the units of the target variable (MW):

(3) $$\mathrm{MAE}=\frac{1}{n}\sum_{i=1}^{n}\left|y_{i}-{\hat{y}}_{i}\right|$$

### 4.2. Feature Importance Analysis with Gradient-Boost-Based Selection

The gradient-boost-based feature selection yields a consistent and interpretable picture of the drivers of short-term load dynamics in the Croatian system.

For the single-model configuration (one model forecasting all 24 h), a total of 41 predictors are retained. The ranking confirms that historical load is the dominant source of information: the load at a lag of 168 h (one week earlier) emerges as the most influential feature, highlighting the strength of weekly consumption patterns. In addition, the selected subset systematically includes the following: short- and medium-term load lags (e.g., 48, 72, 336 h), forecasted temperature and dew point from the TIGGE-based NWP fields for both Zagreb and Split at multiple lead times, and calendar indicators such as holidays and working days. Together, these features underline the combined relevance of recurrent demand structure, meteorological conditions, and calendar effects in shaping the next-day load.

For the 24-model, with its hour-specific configuration, the selected feature sets exhibit expected variability across hours, but with several predictors consistently dominating. The load at a 168 h lag is identified as an important feature in all hourly models and is the top-ranked predictor in more than half of them, reaffirming the central role of weekly patterns. The 336 h lag (two weeks earlier) and 48 h lag (two days earlier) are frequently selected as well, indicating that the models exploit information on multiple temporal scales.

Meteorological predictors derived from TIGGE also appear systematically across hours and locations. Forecasted temperatures for Zagreb and Split at selected lead times (e.g., around 18–36 h, depending on the hour and region) are repeatedly chosen, as are specific dew point forecasts, reflecting the sensitivity of load to both absolute temperature and humidity-related effects. Historical measured temperatures, particularly at a 168 h lag, further contribute as stable predictors in many hourly models.

Calendar-related variables retain a strong and robust influence: the holiday indicator is selected in all 24 hourly models, the working-day indicator in almost all of them, and the month-of-year feature is frequently included, especially for morning and afternoon hours. These results confirm that the gradient-boost-based selection systematically identifies a compact set of predictors that is physically meaningful and consistent with established STLF knowledge, while providing a well-founded input space for the SVR, DNN, and hybrid DNN-SVR models evaluated in this study.

### 4.3. Standalone DNN Baseline: Architecture Search

The proposed hybrid DNN-SVR model is implemented in two stages. First, a standalone feedforward DNN is trained as a nonlinear feature extractor; second, an SVR is fitted on the penultimate-layer representations.

All DNNs are multilayer perceptrons (MLPs) implemented in TensorFlow/Keras [36]. Each network comprises an input normalization layer, fully connected hidden layers with ReLU activations, and a single linear output neuron. Models are trained with the Adam optimizer (learning rate $10^{-3}$) and MAE loss for up to 1000 epochs with early stopping. A chronological 80/20 train–validation split is used; the normalization layer is adapted on the training fold only; mini-batches are shuffled during training; and the epoch with the lowest validation MAE is retained to stabilize training and mitigate overfitting.

To identify the most suitable DNN configuration, we perform a targeted architecture search under this fixed protocol. In the hour-specific setting, a separate DNN is trained for each target hour using only its corresponding samples. Candidate networks vary in both depth and width. For each architecture, we record for every hour the minimum validation MAE and use the average across all 24 h as the selection criterion, reflecting typical generalization over the full daily load profile.

Table 2 presents the results. The architecture with two hidden layers of 256 neurons (256 × 256) achieves the lowest average validation error, indicating superior stability and accuracy across hours. In addition to empirical performance, similar feedforward neural network configurations with two hidden layers and approximately 256 neurons per layer have been reported in the literature for load and energy forecasting tasks, where they were found to provide sufficient capacity for modeling nonlinear relationships among multiple input features [38,39]. From a theoretical perspective, a two-hidden-layer feedforward network with sufficient width constitutes a universal function approximator, offering adequate representational capacity without introducing unnecessary architectural depth [40]. Based on these considerations, the 256 × 256 network is selected as the principal standalone DNN configuration and as the foundation for the DNN-SVR hybrids evaluated later.

### 4.4. Ablation Study: Contribution of the SVR Regression Head

This ablation study isolates the effect of replacing the DNN’s linear output layer with an SVR regression head. We compare two families of predictors: standalone DNN models and the proposed DNN-SVR hybrids, which use the same inputs, training protocol, and hour-specific configuration but replace the final dense layer with an SVR operating on the penultimate-layer representation.

All experiments are carried out using the best standalone DNN architecture identified in the previous subsection (256 × 256). The training procedure, feature set, and hour-specific setup are kept identical; the only modification is the substitution of the output layer by an SVR head (either linear or RBF kernel).

Table 3 reports the aggregated results on the 2022 test set. Attaching an SVR head reduces MAPE relative to the standalone DNN. For the 256 × 256 network, the RBF-based hybrid lowers MAPE from 1.9867% to 1.8950%, a decrease of 0.0917 percentage points (4.62% relative). In the Croatian power system, such incremental accuracy gains can be operationally relevant, as they reduce imbalance volumes and associated costs in a system with notable import dependence and exposure to volatile balancing energy prices [41,42]. Improved short-term forecasts also facilitate more accurate day-ahead scheduling and more efficient procurement of cross-border transmission capacity, thereby reducing reliance on costly intraday adjustments and imbalance settlements [43].

Formal tests corroborate the aggregate gains: a two-sided Diebold-Mariano test on MAE loss rejects the null of equal predictive accuracy in favor of the DNN-SVR (RBF) variant (DM=6.118, $p=9.48\times 10^{-10}$). A paired Wilcoxon test on absolute errors reaches the same conclusion (W=15,488,209, $p=1.003\times 10^{-16}$).

**Figure 4:**
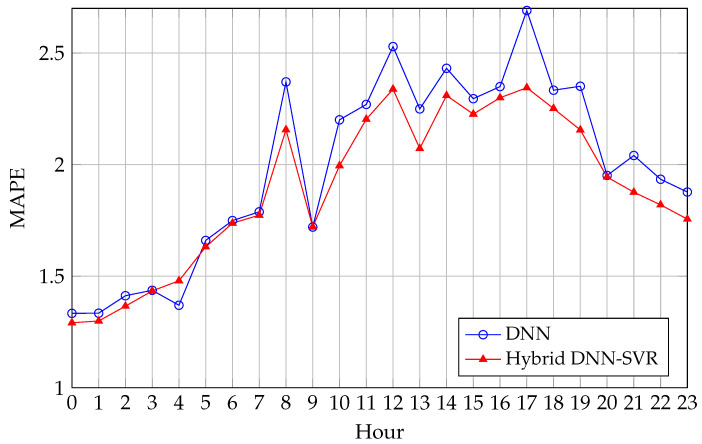
Hour-by-hour MAPE comparison between the standalone DNN and the hybrid DNN-SVR model (RBF kernel) for the 2022 test year.

Figure 4 provides an hour-by-hour view of this effect. For almost all 24 h, the hybrid DNN-SVR model with an RBF kernel attains equal or lower MAPE than the standalone DNN, with the largest relative gains occurring during peak and shoulder periods. The hybrid configuration therefore does not simply improve aggregate error: it consistently tightens the error profile over the entire daily cycle.

Overall, this ablation confirms that the SVR regression head adds measurable value on top of the learned DNN representations. The DNN captures rich nonlinear features from load, weather, and calendar inputs, while the SVR exploits these features through margin-based regression, yielding a more accurate and stable forecasting model.

### 4.5. Statistical Validation with Multi-Seed Experiments

To ensure the reported improvements are not artifacts of random initialization, we conducted 10 independent training runs (seeds 0–9) for both the standalone DNN and hybrid DNN-SVR models using the 256 × 256 architecture and the full 2006–2021 training period. Table 4 presents the aggregated results.

Statistical testing confirms the significance of the improvement. A paired *t*-test yields t=15.80 with p<0.000000021, and Cohen’s d=4.76 indicates a very large effect size. These results demonstrate that the DNN-SVR hybrid consistently outperforms the standalone DNN across different random initializations, and the improvement is highly statistically significant.

### 4.6. Impact of Data Size on Forecasting Performance

To evaluate how the amount of historical data influences forecasting accuracy, both the standalone DNN and the hybrid DNN-SVR models were trained on datasets covering different time spans. Each dataset included data from the specified starting year up to the end of 2021, while the year 2022 was reserved exclusively for model testing. Within each training set, 20% of the samples were used for validation to prevent overfitting and ensure consistent performance comparison.

Table 5 presents the obtained results. A clear pattern emerges: for the hybrid DNN–SVR model, accuracy generally improves as the training window expands from 2018 to 2021 to longer histories, reaching the lowest MAPE of 1.8950 on the 2006–2021 dataset (with 2014–2021 virtually tied at 1.8968 and 2012–2021 slightly higher at 1.9059). For the pure DNN model, the best performance is achieved on the 2014–2021 dataset (MAPE = 1.9786), with slightly higher errors for both shorter and longer horizons. Across all configurations, the hybrid model consistently outperforms the standalone DNN, confirming the advantage of integrating nonlinear SVR regression with deep feature representations.

**Figure 5:**
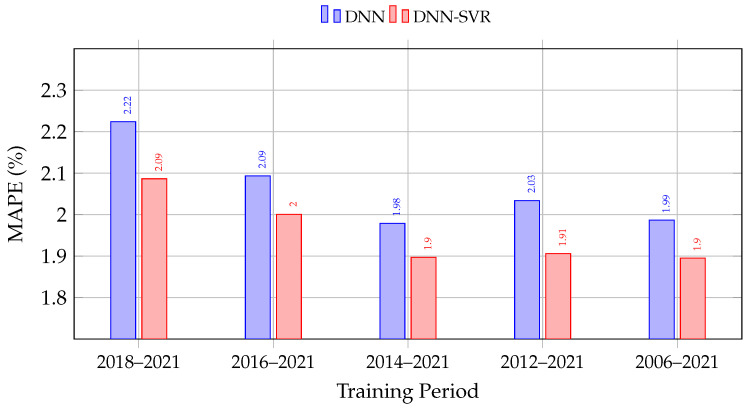
MAPE comparison of DNN and hybrid DNN-SVR models across different training periods. The hybrid model consistently achieves lower error, with the best performance (1.895%) on the full 2006–2021 training set.

Figure 5 provides a visual summary of the MAPE trends across training periods. As the training window expands, both models improve, with the hybrid DNN-SVR consistently outperforming the standalone DNN by 4–7% relative across all periods.

**Figure 6:**
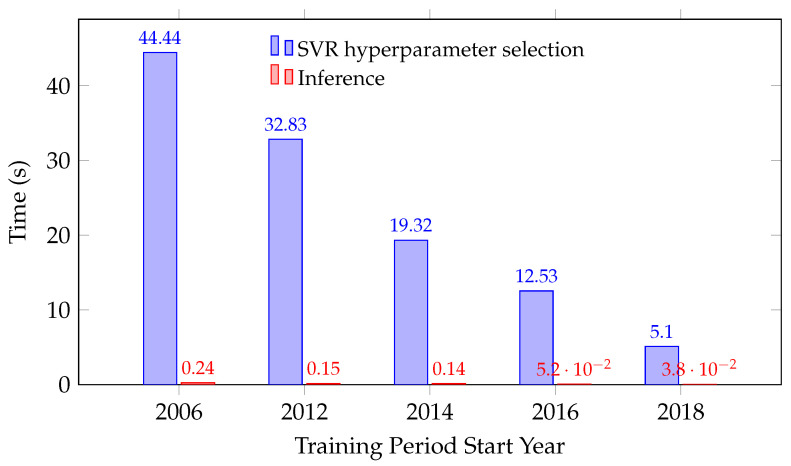
SVR hyperparameter selection time and inference time vs. training period start year (hour-specific model; first of 24; validation 20%).

The hybrid DNN-SVR pipeline introduces a modest additional offline cost for SVR hyperparameter selection, while online inference remains fast. Figure 6 shows the time to select SVR hyperparameters (on the development data tail) and the execution time to generate predictions for the entire 2022 test year for a single target hour (*the first of the 24 h specific models*). As the training period extends backward (i.e., more data), the tuning time increases, whereas inference latency stays well below one second, indicating that the hybrid’s added cost is operationally acceptable when retraining is infrequent and prediction speed is critical.

### 4.7. Benefits of Hour-Specific Submodels

In addition to the architectural choices discussed above, we systematically compared two learning strategies that are frequently contrasted in the STLF literature: training 24 separate hour-specific models (1 for each target hour) and training a single consolidated model that predicts all 24 h simultaneously, with the hour-of-day included as an input feature. The comparison is aligned across models, feature sets, and training procedures for the two considered architectures: a standalone feedforward DNN and the hybrid DNN-SVR. For each configuration, hyperparameters are tuned on the development period using a fixed validation split of 20%, and final performance is reported on the held-out test year 2022.

Table 6 reports the resulting MAPE values. Across both architectures, the hour-specific submodels consistently outperform the single global model, despite being trained on fewer samples per model.

On average, the hour-specific submodels reduce MAPE by about 18% relative to the single-model alternative across both architectures. In the hybrid setting, the hour-specific approach attains 1.8950% MAPE versus 2.3159% for the unified hybrid model (an 18.2% relative reduction). The hour-specific DNN (1.9867%) likewise improves upon the unified DNN (2.4114%), corresponding to a 17.6% reduction.

These results indicate that decomposing the forecasting task by hour is an effective strategy to reduce the heterogeneity of the learning problem. Hour-specific models can adapt to distinct intraday regimes (e.g., sharp morning ramps, afternoon plateaus, low and less weather-sensitive night-time demand), whereas a single consolidated model must simultaneously fit markedly different distributions and load–weather relationships across all hours. Consequently, despite having access to a larger overall sample size, the single model exhibits higher average error than the strategy with specialized hourly models.

### 4.8. Impact of Global Weather Data Integration

This subsection quantifies the effect of incorporating global NWP-based weather forecasts into the hybrid DNN-SVR models. The comparison is performed between models that use only historical load and local weather observations, and models that additionally include TIGGE-derived forecast variables for Zagreb and Split as exogenous inputs. The analysis is carried out for both a single-model strategy (one model for all 24 h) and a 24-model hour-specific strategy.

Table 7 summarizes the results. In both configurations, adding NWP-based meteorological predictors substantially reduces the forecasting error. For the single hybrid model, MAPE falls from 3.1332% to 2.3159%, a 26.09% relative reduction. The effect is also pronounced for the hour-specific hybrid framework, where MAPE drops from 2.6760% to 1.8950% (a 29.19% relative reduction).

**Figure 7:**
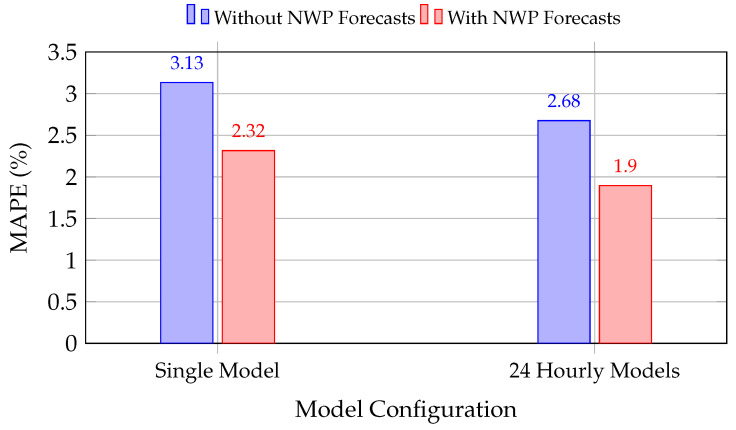
Impact of NWP-based weather forecasts on hybrid DNN-SVR performance. Both model configurations benefit substantially from forecast integration, with the 24 hourly models achieving the best overall accuracy (1.895% MAPE).

Figure 7 visualizes these improvements. The addition of NWP forecasts yields substantial error reductions for both modeling strategies, with the 24 hourly models achieving the lowest overall MAPE (1.895%) when combined with forecast data.

**Figure 8:**
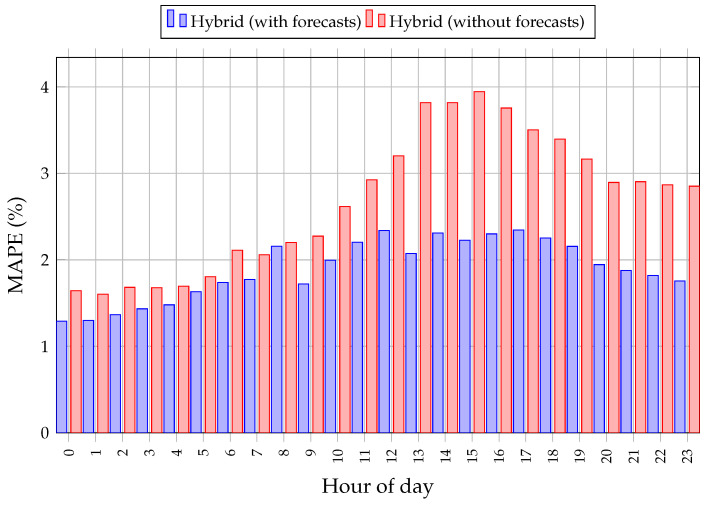
Hourly MAPE comparison for the 24-model hybrid DNN-SVR framework with and without forecast-based weather predictors.

The hourly error profiles in Figure 8 further illustrate these gains for the 24-model hybrid strategy. Improvements are observed across all hours, with the largest relative reductions in the early afternoon and early evening, when load is more sensitive to temperature and weather-driven behaviour. Smaller but still consistent benefits are seen during the late night and early morning hours, where demand is less weather-dependent.

Overall, these results provide clear empirical evidence that integrating global NWP-based forecasts, when properly aligned and exploited in an hour-specific hybrid architecture, substantially enhances day-ahead STLF performance for the Croatian power system.

### 4.9. Comparison with LSTM Seq2Seq + Attention Models

As a recurrent deep learning baseline, we employ an LSTM Seq2Seq architecture with attention [44,45]. The model uses the same feature set as other approaches: a 24 h historical window (load, temperature, calendar features with sine/cosine encodings) and a 39 h known-future horizon (NWP forecasts, calendar indicators). The encoder–decoder architecture (128 units per layer) with attention over encoder outputs produces 24 h day-ahead forecasts. Training uses the Adam optimizer (learning rate $10^{-3}$), MAE loss, and batch size 32, with early stopping (patience 20) and learning-rate reduction on plateau (factor 0.5, patience 7, min LR $10^{-5}$).

To quantify sensitivity to the amount and recency of training data, the Seq2Seq + Attention model is trained on multiple historical windows using the same validation protocol. The results in Table 8 show that a medium-length window (2012–2021) offers the best trade-off between data volume and representativeness, whereas including very old data or using only short recent segments leads to degraded performance.

Overall, the LSTM Seq2Seq + Attention model provides a strong and conceptually modern baseline that effectively captures temporal structure and benefits from high-quality recent data. However, under the same training and evaluation conditions, it is consistently outperformed by the hour-specific hybrid DNN-SVR framework, which achieves lower MAPE on the national-scale Croatian STLF task.

### 4.10. Comparison with Transformer-Based Models

Transformer architectures, originally proposed for natural language processing [19], have become a strong option for time-series forecasting due to their ability to model long-range dependencies via self-attention and to process sequences in parallel. In the context of STLF, these properties are attractive for multi-horizon day-ahead forecasting and for capturing complex interactions between load, weather, and calendar effects. To position the proposed hybrid DNN-SVR approach against modern sequence models, we implement several Transformer-based baselines under the same experimental protocol (common feature set, chronological 80/20 validation split on the development period, evaluation on the held-out test year):

- An encoder-only Transformer for hour-specific prediction with a dense regression head;
- A two-stage hybrid model combining a Transformer encoder backbone with an SVR regression head;
- A Temporal Fusion Transformer (TFT) [46] designed for multi-horizon forecasting;
- A PatchTFT model [24] based on patch-wise self-attention for univariate and multivariate time-series forecasting.

All Transformer-based baselines use the same curated feature space as the hybrid DNN-SVR approach: historical load, observed near-surface temperature from Zagreb and Split, calendar and sinusoidally encoded time features, and a selected subset of TIGGE-based NWP forecast variables, where the NWP inputs are restricted to those identified as relevant by the gradient-boost feature selection procedure.

#### 4.10.1. Encoder-Only Transformer (Model 1)

The encoder-only Transformer operates in an hour-specific setup with 24 h input windows. Each model uses two encoder blocks with multi-head self-attention (four heads, hidden size 64), producing hour-ahead forecasts via a dense projection layer. Hour-specific NWP feature subsets are selected via gradient boosting. Training uses Adam ($10^{-4}$), MAE loss, 80/20 validation split, and early stopping (patience 20).

#### 4.10.2. Transformer Encoder + SVR Hybrid (Model 2)

To examine whether SVR benefits Transformer-based representations, we construct a two-stage hybrid: (1) train the Transformer encoder from Model 1 with a temporary dense head, (2) extract penultimate-layer embeddings, (3) fit an RBF-SVR on the embeddings via grid search over (C,ϵ,γ) using the most recent 3 years of data, and (4) pair the frozen backbone with the tuned SVR for inference. This procedure is repeated for all 24 h. The hybrid achieves lower MAPE than the dense-head Transformer, confirming that kernel-based regression effectively exploits Transformer-derived features.

#### 4.10.3. Temporal Fusion Transformer (Model 3)

The TFT [46] is a multi-horizon forecasting model combining LSTM encoder–decoder layers (64 hidden units), Variable Selection Networks for feature weighting, and Gated Residual Networks. It uses the same two-stream input as LSTM and PatchTFT: a 24 h historical context and a 39 h known-future sequence. Training uses Adam ($10^{-3}$), batch size 32, early stopping (patience 20), and ReduceLROnPlateau scheduling (factor 0.5, patience 5, min LR $10^{-5}$).

#### 4.10.4. PatchTFT (Model 4)

PatchTFT [24] segments time series into patches before applying self-attention, capturing local patterns more effectively. It uses the same two-stream input as TFT: a 24 h historical window and a 39 h known-future sequence. The encoder operates on overlapping patches (length 12, stride 6), while the decoder applies cross-attention between known-future inputs and encoded patch representations. Training uses Adam ($10^{-3}$), MAE loss, batch size 32, early stopping (patience 20), and learning-rate scheduling (factor 0.5, patience 5, min LR $10^{-5}$).

#### 4.10.5. Input Features and Stream Design Across Models

The PatchTFT, LSTM Seq2Seq + Attention, and TFT baselines adopt a consistent two-stream input structure. The past observation stream consists of a 24 h historical window comprising past load, observed near-surface temperatures from Zagreb and Split, holiday indicators, and six periodic encodings derived from calendar variables, yielding 10 features in total.

The known-future stream spans a 39 h forecast horizon and includes calendar indicators, periodic time encodings, holiday flags, and a subset of TIGGE-based NWP variables. These NWP features are selected from an original pool of 364 raw candidates using Gradient Boosted Trees, as detailed in Section 3.1 and Section 3.3. In our case, the resulting feature set includes 31 NWP variables, representing horizon-invariant meteorological predictors across two cities (Zagreb and Split), including surface temperature (2t), dew point (2d), and other atmospheric quantities sampled at multiple lead times. The resulting known-future input tensor comprises 37 features per time step.

In contrast, the Encoder-Only Transformer (Model 1) and the Transformer + SVR hybrid (Model 3) operate on a single-stream input structure. This stream aggregates a 24 h lookback window of past load, holiday indicators, six periodic time encodings, and a tailored subset of hour-specific NWP features obtained via Gradient Boosted Trees, as described in Section 3.1. Feature selection is performed independently for each of the 24 forecast hours, enabling hour-specialized predictors for both the dense-head Transformer and the SVR regression stages.

These curated input pipelines ensure that all Transformer-based models operate under consistent temporal and covariate protocols. While time-series baselines apply feature selection solely to the known-future NWP components, the DNN-SVR regression model leverages a flat feature vector composed of all selected predictors, including both lag-based and forecast-derived components. This architecture-aware setup enables fair and reproducible comparisons across model families.

#### 4.10.6. Overall Comparison

Table 9 reports the MAPE for the examined Transformer-based approaches (24-model hour-specific setups and multi-horizon models, as applicable). The encoder-only Transformer with shared-window training already performs competitively; replacing the dense head with an SVR regression stage (Model 2) further reduces the error to 2.1012%, outperforming all purely Transformer-based variants and TFT in this study. PatchTFT (Model 4) achieves 2.1904% MAPE, underperforming the proposed DNN-SVR hybrid (1.895%) by approximately 13.5%.

#### 4.10.7. Effect of Training Set Size

Table 10 reports the effect of training-window length on TFT. TFT benefits from longer and more diverse histories: enlarging the window systematically reduces error and makes the model less sensitive to the exclusion of older data.

Taken together, these results show that well-designed Transformer models are competitive multi-horizon forecasters, attaching an SVR head to Transformer embeddings yields additional gains and reinforcing the usefulness of kernel regression on learned feature spaces; however; the hour-specific hybrid DNN-SVR framework still attains the lowest MAPE overall in our experiments. This supports the central claim that structured hybridization of deep feature extractors with SVR, combined with hour-specific modeling and enriched NWP-based inputs, provides a particularly effective framework for operational STLF in the Croatian power system.

### 4.11. Baseline Model Hyperparameters

Table 11 summarizes the hyperparameter configurations used for the baseline deep learning models, including PatchTFT [24] and the SVR component. All neural network models were tuned using the 2021 validation set, with the same data splits applied consistently across architectures.

## 5. Discussion

This study examined the effectiveness of an hour-specific hybrid DNN-SVR framework for national-scale Short-Term Load Forecasting (STLF) and analyzed its behaviour in relation to established baselines and key design choices. Rather than introducing SVR merely as an alternative output layer, the work combined several elements that are rarely addressed jointly: a 17-year Croatian load dataset with a dedicated test year, a set of 24 specialized hybrid models trained on hour-specific representations, a systematic gradient-boost-based feature selection pipeline, integration of global NWP (TIGGE) data into the predictor set, and a unified comparison against modern deep learning architectures.

The results consistently indicate that this structured hybridization is beneficial in the considered setting. The DNN components act as nonlinear feature extractors tailored to individual hours, while the SVR heads, particularly with RBF kernels, exploit these compact representations to achieve lower forecast errors than both standalone DNNs and standalone SVR models. When contrasted with LSTM and Transformer-based baselines implemented under the same data and evaluation protocol, the hybrid framework attains competitive or superior accuracy, especially on the held-out 2022 test set, thereby reinforcing the practical value of the proposed design.

### 5.1. The Synergy of DNN and SVR

The primary success of the hybrid model lies in the synergistic combination of its two components. The DNN acts as a powerful, non-linear feature extractor, learning high-level representations from the raw input data (historical load, weather, and temporal features). The SVR, particularly with an RBF kernel, then excels as a robust regression head, operating on this refined feature space to produce the final forecast. This two-stage process leverages the strengths of both methods: the deep learning component captures complex patterns that might be missed by a standalone SVR, while the SVR provides a precise regression boundary that can be more effective than a simple dense output layer in a DNN. For the best-performing architecture, the hybrid DNN-SVR (1.8950% MAPE) achieved a 4.62% relative error reduction compared to its standalone DNN counterpart (1.9867% MAPE), quantifying the practical value of the SVR head.

An interesting observation is that the DNN-SVR hybrid (1.8950% MAPE) outperforms the Transformer + SVR hybrid (2.1012% MAPE) despite both employing the same SVR regression head. We hypothesize that this difference arises from the nature of the representations and input design. Transformer encoders with self-attention produce contextually weighted features that may be more sparse or distributed than the dense activations from a standard feedforward DNN. The DNN’s fully connected layers create more uniform, dense feature representations that are better suited for SVR’s RBF kernel-based regression. Additionally, the Transformer model processes a 24 h historical window including load, observed temperature, calendar features, and cyclic encodings, with feature selection applied only to NWP forecast features. In contrast, the hour-conditioned DNN receives a more curated input with 30–40 XGBoost-selected features per hour, resulting in a more compact feature space optimized for the specific forecasting task.

Finally, regarding the training strategy, this study deliberately employs a decoupled two-stage approach rather than joint end-to-end optimization. While differentiable SVM layers exist, the two-stage protocol offers distinct operational advantages in this context. First, it ensures stability: SVR optimization relies on a fixed feature space, and simultaneously updating the deep feature extractor and the kernel margin can lead to convergence instabilities. Second, it enhances interpretability: by freezing the DNN parameters, the marginal contribution of the SVR head can be rigorously isolated and quantified (as evidenced in the ablation study), ensuring that the performance gains are attributable to the margin-based regression rather than gradient interactions.

### 5.2. The Critical Role of Modeling Strategy

A significant finding is the performance difference between modeling strategies. For the DNN-based models, a multi-model strategy using 24 separate models, each trained on hour-specific data, yielded substantially better accuracy than a single, generalized model or a multi-model approach using a comprehensive dataset. This suggests that for feedforward networks, specializing the model to the unique load patterns of each hour is more effective than attempting to generalize across the entire day.

### 5.3. Impact of Data Volume and External Data Integration

The experiments confirmed that model performance is highly correlated with the volume of training data. Longer historical datasets consistently led to more accurate forecasts, though with a significant trade-off in increased computational time, especially for the SVR component of the hybrid model.

Furthermore, integrating global numerical weather prediction forecasts proved critical for high accuracy: adding these prognostic weather features markedly improved performance across all configurations, with the most pronounced gains observed in the hour-specific multi-model strategy.

### 5.4. Heterogeneous Sensor Data Fusion Perspective

Viewed from a sensing perspective, the empirical results presented in Section 4 indicate that the performance gains associated with the proposed framework arise from the effective exploitation of heterogeneous information sources rather than from increased model complexity alone. In particular, the substantial reduction in forecasting error observed when global NWP-based predictors are incorporated suggests that these inputs provide information that is complementary to, rather than redundant with, historical load and local meteorological measurements.

The results in Table 7 and Figure 7 demonstrate that forecast-based weather information contributes most strongly in the hour-specific hybrid configuration, where each model is trained to capture distinct intraday load–weather relationships. This finding supports the interpretation that global NWP forecasts function as physically consistent, forward-looking virtual sensors that enhance the model’s situational awareness under conditions where historical measurements alone are insufficient, such as during rapidly changing weather regimes or atypical demand patterns.

Importantly, the robustness of the observed gains across all hours, as shown in Figure 8, indicates that the benefits of heterogeneous data integration persist across long-term operational conditions in which local meteorological observations may exhibit temporal irregularities or limited availability. This suggests that the hybrid framework does not rely on any single sensing modality, but instead leverages dataset-level redundancy between data sources to stabilize predictions under realistic operational constraints. From this perspective, the improved accuracy achieved by the proposed DNN-SVR model reflects the effective utilization of complementary sensor information within an hour-specific learning strategy, rather than a simple increase in input dimensionality.

Overall, these observations position the proposed forecasting framework as an example of how heterogeneous sensor information can be exploited in data-driven energy systems, where predictive performance emerges from the alignment of model structure with the informational characteristics of diverse data sources.

### 5.5. Practical Implications and Computational Considerations

From a practical standpoint, the proposed hybrid DNN-SVR model, deployed in a multi-model, hour-specific strategy, offers a clear path to improving forecasting accuracy for grid operators. The practical benefit of this approach was validated in the benchmark comparison, where the proposed model (1.8950% MAPE) achieved a 9.8% relative error reduction compared to the next-best baseline, the Transformer + SVR (2.1012% MAPE), a 13.5% reduction against the PatchTFT (2.1904% MAPE), a 14.6% reduction against the Temporal Fusion Transformer (2.2199% MAPE), and a 21.4% reduction relative to an LSTM Seq2Seq + Attention baseline (2.41% MAPE). This significant performance gap demonstrates the value of the hour-specific hybrid framework for operational STLF in power system management. The improved accuracy can translate directly to better unit commitment decisions, reduced reserve requirements, and more efficient grid operations, particularly in systems integrating variable renewable energy sources.

The primary operational consideration is computational cost. Training 24 separate hybrid DNN-SVR models requires more resources than a single unified model. However, this cost is acceptable in operational settings where models are retrained infrequently (e.g., monthly or quarterly), and the accuracy gains justify the additional computational investment. In terms of total training duration on the specified hardware, the complete set of 24 h specific hybrid models requires approximately 60–75 min (including the necessary SVR hyperparameter tuning), which is comparable to the 45–75 min required to train the Temporal Fusion Transformer baseline, and significantly faster than the 2–3 h typically required for the more complex LSTM Seq2Seq architectures. For applications requiring very frequent retraining, alternative architectures may offer different accuracy–efficiency trade-offs.

### 5.6. Generalizability and Scalability

While validated on Croatian data, the proposed framework is designed with transferability in mind. The hour-specific decomposition strategy is applicable to any power system exhibiting diurnal demand patterns, which encompasses virtually all grid systems worldwide. The feature engineering approach leverages standard meteorological variables (temperature, humidity, pressure, wind) available globally through the TIGGE archive, and the model architecture contains no Croatia-specific components—only historical load and weather data are required. For other European countries, the ENTSO-E Transparency Platform provides comparable load data, enabling direct application of this methodology.

For larger multi-regional or continental-scale systems, the 24-model architecture scales linearly: *N* regions would require 24N models, all of which can be trained and deployed in parallel on modern GPU infrastructure. The main adaptation for new regions would be retraining to capture region-specific demand patterns (e.g., heating-dominated vs. cooling-dominated climates, distinct industrial schedules). For hierarchical forecasting across interconnected regions, the framework could be extended by adding spatial aggregation layers that combine regional DNN-SVR forecasts while respecting inter-regional power flows—a promising direction for future work.

Looking ahead, an important extension is the integration of this forecasting framework into decision-support pipelines for economic dispatch and unit commitment. Recent work on value-oriented and cost-oriented forecasting [47,48] has shown that prediction accuracy alone may not guarantee optimal operational outcomes; forecasts optimized for operational cost rather than pure accuracy can yield better economic performance. Extending the proposed framework to incorporate cost-sensitive objectives, where the loss function reflects the asymmetric costs of over- and under-prediction in power system operations, represents a promising direction for future research.

## 6. Conclusions

This work presented and evaluated an hour-specific hybrid DNN-SVR framework for day-ahead short-term load forecasting, tested on 17 years of Croatian national electrical load data within a rigorous experimental protocol. The primary contribution is not the hybridization concept itself, but rather a comprehensive, empirically validated integration of multiple design elements: hour-specific modeling, systematic feature engineering, global weather data assimilation, and extensive baseline comparisons that collectively demonstrate how hybrid architectures can be effectively deployed for operational STLF in real-world power systems.

The key findings and practical insights are as follows:

1. **Hybrid architecture effectiveness:** Integrating an SVR regression head on DNN-extracted features consistently reduces the forecasting error compared with a standalone DNN. The tested configuration achieved a 4.62% relative reduction in MAPE (from 1.9867% to 1.8950%), confirming that margin-based regression on learned representations yields tangible accuracy gains;
2. **Hour-specific modeling strategy:** Decomposing the 24 h forecasting problem into specialized hourly models proved essential for feedforward and hybrid architectures, reducing error by approximately 18.2% compared to a single unified model. This strategy enables each model to capture distinct intraday load regimes without forcing a common representation across heterogeneous hourly patterns, at the cost of increased computational requirements;
3. **Critical role of weather data:** Integrating TIGGE-based global NWP forecasts with local meteorological observations yielded a 29.2% accuracy improvement for the hour-specific hybrid framework, demonstrating that high-quality prognostic weather data is not merely beneficial but essential for achieving state-of-the-art STLF performance in weather-sensitive power systems;
4. **Training data volume vs. computational cost:** Longer historical training periods systematically improve accuracy, with the 2006–2021 window achieving the lowest test error (1.8950% MAPE).
5. **Competitive advantage over modern baselines:** Under identical experimental conditions (same data, features, preprocessing, and evaluation protocol), the proposed hour-specific hybrid framework (1.8950% MAPE) outperformed all evaluated baselines on the held-out 2022 test set, including LSTM Seq2Seq + Attention (2.41% MAPE; 21.4% relative improvement), the Temporal Fusion Transformer (2.2199% MAPE; 14.6%), the PatchTFT (2.1904% MAPE; 13.5%), and the Transformer + SVR hybrid (2.1012% MAPE; 9.8%). This systematic comparison establishes the hybrid DNN-SVR approach as a strong benchmark for national-scale STLF.

For power system operators, these results suggest that the additional complexity of maintaining 24 h specific hybrid models is justified by the substantial accuracy gains, particularly when reliable weather forecasts are available and retraining frequency is moderate (e.g., monthly or quarterly updates). The framework is immediately applicable to similar national or regional STLF problems with comparable data availability.

From a broader sensing perspective, this study shows that short-term load forecasting can be viewed as a heterogeneous sensor data fusion problem. The proposed framework integrates grid-level load measurements, local meteorological observations, and global numerical weather prediction outputs. Although all inputs are aligned to an hourly forecasting task, the NWP predictors retain an inherent temporal structure through multiple forecast lead times, providing forward-looking information distinct from retrospective measurements. By adopting a conservative, feature-level fusion strategy that preserves temporal integrity and data quality, the approach reflects realistic constraints encountered in long-term sensor-driven applications.

In summary, this study demonstrates that carefully designed hybrid DNN-SVR frameworks, when combined with hour-specific modeling strategies and comprehensive weather data integration, provide a practical and effective solution for national-scale short-term load forecasting that outperforms current state-of-the-art deep learning approaches.

## Figures and Tables

**Table 1:** Summary of input feature categories before XGBoost selection.

Category	Count	Description
TIGGE NWP forecasts	364	2 cities × 2 grid points × 7 variables × 13 leads
Load lags	39	33 near-term + 6 weekly anchors
Temperature lags	76	2 cities × (33 near-term + 5 anchors)
Calendar	3	Holiday type, weekday, month
**Total**	**482**	Before per-hour feature selection

**Table 2:** DNN network structures and average minimum validation MAE (hour-specific multi-model setup).

DNN Network Structure ^a^	Average Min Val MAE
**256 × 256**	**37.215**
128 × 128 × 128	37.601
64 × 64 × 32	37.776
128 × 128	37.783
64 × 64	38.539
32 × 32	39.368

^a^ Number of neurons in each hidden layer, separated by “×”.

**Table 3:** Ablation study: comparison of standalone DNN and DNN-SVR with RBF kernel for the 256 × 256 network architecture (hour-specific multi-model setup, 2022 test set). All models use identical feature sets obtained via gradient-boost-based selection. Metrics reported: MAPE (%) and MAE.

DNN Network Structure ^a^	DNN		DNN-SVR (RBF)	
	MAPE	MAE	MAPE	MAE
256 × 256	1.9867	42.14	**1.8950**	**40.14**

^a^ Number of neurons in each hidden layer, separated by “×”.

**Table 4:** Multi-seed validation results (10 runs, seeds 0–9) for DNN and DNN-SVR models.

Model	Mean MAPE (%)	Std Dev	95% CI
DNN	1.9560	0.0341	[1.9331, 1.9790]
DNN-SVR	1.8757	0.0210	[1.8616, 1.8899]

**Table 5:** Comparison of DNN and hybrid DNN-SVR models over different training periods (testing year: 2022, validation: 20%).

Training Period (Years)	Model	Training Data Size	Training Time (s)	Execution Time (s)	MAPE
2006–2021	DNN	5476	339.00	0.43	1.9867
2006–2021	DNN-SVR	5476	5480.90	0.67	**1.8950**
2012–2021	DNN	3639	164.92	0.25	2.0338
2012–2021	DNN-SVR	3639	2198.00	0.27	1.9059
2014–2021	DNN	2922	116.17	0.26	1.9786
2014–2021	DNN-SVR	2922	1373.78	0.20	1.8968
2016–2021	DNN	2182	91.62	0.20	2.0932
2016–2021	DNN-SVR	2182	709.91	0.13	2.0006
2018–2021	DNN	1461	83.00	0.19	2.2238
2018–2021	DNN-SVR	1461	291.68	0.11	2.0863

**Table 6:** Comparison of hour-specific submodels (24 models) vs. single consolidated model for DNN and hybrid DNN-SVR.

Approach	Total DNN Samples ^a^	DNN MAPE	DNN-SVR MAPE
24 h specific models	5476	1.9867	1.8950
Single consolidated model	130,721	2.4114	2.3159

^a^ Number of training samples per configuration; all models use a 20% validation split on the development period and are evaluated on the 2022 test set.

**Table 7:** Impact of forecast-based weather predictors on hybrid DNN-SVR performance.

Model	Without Forecasts (MAPE)	With Forecasts (MAPE)	Relative Reduction (%)
Single hybrid model (24 h)	3.1332	2.3159	26.09
24 hourly hybrid models	2.6760	1.8950	29.19

**Table 8:** Impact of training period on LSTM Seq2Seq + Attention performance.

Training Period	Training Samples	MAPE (%)	MAE
2006–2021	106,881	2.41	51.11
2012–2021	79,066	2.21	46.26
2018–2021	33,082	3.22	65.53

**Table 9:** Comparison of Transformer-based configurations and training strategies.

Configuration/Strategy	MAPE (%)	MAE (MW)
24 encoder-only Transformers + SVR	**2.1012**	**43.51**
PatchTFT	2.1904	46.00
Temporal Fusion Transformer (TFT)	2.2199	46.65
24 encoder-only Transformers, shared-window training (dense head)	2.4355	47.47

**Table 10:** Impact of training period on TFT performance.

Training Period	Samples	MAPE (%)	MAE
2006–2021	106,881	2.2199	46.65
2012–2021	79,066	2.2299	46.36
2018–2021	33,082	2.7760	56.35

**Table 11:** Hyperparameter configurations for baseline models.

Model	Parameter	Search Range	Selected
LSTM Seq2Seq + Attention	Hidden units	{32, 64, 128, 256}	128
	Layers	{1, 2, 3}	2
	Dropout	{0.0, 0.1}	0.0
Encoder-Only Transformer (Model 1)	Hidden size	{32, 64, 128}	64
	Attention heads	{2, 4}	4
	Layers	{2, 3}	2
	Feed-forward dim	{64}	64
	Dropout	{0.0, 0.1}	0.0
Transformer + SVR Hybrid (Model 2)	Hidden size	64	64
	Attention heads	4	4
	Layers	2	2
	*C*	{16, 32, 64, 128, 256}	Hour-specific
	γ	{scale, auto}	Hour-specific
	ε	{0.05, 0.10, 0.20}	Hour-specific
TFT (Model 3)	Hidden size ($d_{\mathrm{model}}$)	{32, 64, 128, 256}	64
	Attention heads	{2, 4, 8}	4
	Dropout	{0.0, 0.1}	0.0
PatchTFT (Model 4)	Hidden size ($d_{\mathrm{model}}$)	{64, 128}	64
	Attention heads	4	4
	Layers	{2, 3}	2
	Feed-forward dim	{128, 256}	128
	Patch length	12	12
	Patch stride	6	6
DNN-SVR Hybrid	Architecture	Table 2	256 × 256
	*C*	{32, 128, 512}	Hour-specific
	γ	{scale, auto}	Hour-specific
	ε	{0.05, 0.10}	Hour-specific

Notes: “Hour-specific” indicates that a separate model (with independently tuned hyperparameters) is trained for each hour of the day (24 models in total). The DNN architecture “256 × 256” denotes two fully connected hidden layers with 256 neurons each. *C*, γ, and ε are SVR hyperparameters (regularization coefficient, kernel scale, and epsilon-insensitivity, respectively), each tuned (via grid search) for the RBF kernel. The values “scale” and “auto” are set γ as 1/(number of features × Var(*X*)) and 1/number of features, respectively. For both the DNN-SVR and Transformer + SVR hybrids, the optimal γ setting varies across hours, with “auto” being the predominant choice; in the DNN-SVR hybrid, 15 out of 24 h specific models selected “auto”, while 9 selected “scale”.

## Data Availability

The electrical load data used in this study are publicly available from the ENTSO-E Transparency Platform (https://transparency.entsoe.eu/, (accessed on 27 January 2023)). Global numerical weather prediction forecasts were obtained from the TIGGE archive (https://apps.ecmwf.int/datasets/data/tigge/, (accessed on 27 January 2023)), which requires registration but is freely accessible for research purposes. Historical local meteorological observations were sourced from Weather Underground (https://www.wunderground.com/history/, (accessed on 27 January 2023)). All models are implemented using standard open-source libraries: TensorFlow [35] and Keras [36] for deep learning, scikit-learn for SVR, and XGBoost for feature selection.

## Abbreviations

The following abbreviations are used in this manuscript:

STLF	Short-Term Load Forecasting
DNN	Deep Neural Network
SVR	Support Vector Regression
SVM	Support Vector Machine
NWP	Numerical Weather Prediction
TIGGE	The International Grand Global Ensemble
LSTM	Long Short-Term Memory
TFT	Temporal Fusion Transformer
MAE	Mean Absolute Error
MAPE	Mean Absolute Percentage Error
RBF	Radial Basis Function
ReLU	Rectified Linear Unit
MLP	Multilayer Perceptron
XGBoost	Extreme Gradient Boosting
FS	Feature Selection
ARIMA	Autoregressive Integrated Moving Average
Seq2Seq	Sequence-to-Sequence
VSN	Variable Selection Networks
PatchTFT	Channel-independent patch time series Transformer
